# An epidemiological investigation of porcine circovirus 3 infection in cattle in Shandong province, China

**DOI:** 10.1186/s12917-019-1793-0

**Published:** 2019-02-13

**Authors:** Wei Wang, Wenchao Sun, Liang Cao, Min Zheng, Yilong Zhu, Wenjie Li, Cunxia Liu, Xinyu Zhuang, Jialiang Xing, Huijun Lu, Tingrong Luo, Ningyi Jin

**Affiliations:** 1 College of Animal Science and Technology, Guangxi University, Nanning, 530004 China; 2 Institute of Virology, Wenzhou University, Wenzhou, 325035 China; 3 Institute of Military Veterinary Medicine, Academy of Military Sciences, Changchun, 130122 China; 4 Academy of Military Sciences, Institute of Military Veterinary, Guangxi Center for Animal Disease Control and Prevention, Nanning, China; 5 Institute of Poultry Science, Shandong Academy of Agricultural Sciences, Jinan, Shandong China

**Keywords:** Porcine circovirus type 3, Bovine, Epidemiology, Phylogenetic analysis

## Abstract

**Background:**

Porcine circovirus type 3 (PCV3) is a single-stranded, closed circular DNA virus, which causes porcine dermatitis and nephropathy syndrome (PDNS), multisystemic inflammation, and reproductive failure. The present study aimed to investigate the seroprevalence of PCV3 in cattle (*Bos taurus*) in Shandong province, China, and examine its genome diversity.

**Results:**

PCR amplification and sequencing showed that 74 of 213 bovine samples (34.7%) were positive for PCV3. Among them, the capsid gene (*n* = 12) and the complete genome (*n* = 4) were sequenced. These sequences had high identities to the reference capsid gene (98.0–100%) and the complete genome (97.5–99.8%). The PCV3 strains were classified into two different genotypes (PCV3a and PCV3b), according to phylogenetic analysis based on the complete genome and capsid gene sequences. Specifically, the bovine-origin strains in this study were grouped into PCV3a, showing a close relationship with PCV3-US/SD2016 (American strain; GenBank: KX966193.1). Notably, a comparison of the inferred amino acid sequences revealed a mutation from D124 to Y124.

**Conclusion:**

This was the first seroprevalence and genetic investigation of PCV3 in cattle in Shandong province, China. The results could provide insights into the epidemiology and pathogenesis of this important virus.

## Background

Porcine circovirus (PCV; *Circoviru*s, *Circoviridae*) is a single-stranded, non-enveloped, closed circular DNA virus [1, 2]. PCV has been reported as one of the smallest viruses. Its genome contains two major open reading frames (ORF1 and ORF2), encoding a replication-associated protein (Rep) and capsid protein (Cap), respectively [3]. Specifically, Cap is a major structural protein, containing a number of cell epitopes that are associated with virus neutralization [4, 5].

Two major genotypes of PCV have been reported. Although Porcine circovirus type 1 (PCV1) is considered non-pathogenic, Porcine circovirus type 2 (PCV2) was recognized as one of the main pathogen in PCV-associated disease (PCVAD) [6, 7]. Clinically, infection by PCV2 causes systemic, respiratory, and enteric manifestations in pigs, such as post-weaning multi-systemic wasting syndrome (PMWS) and PDNS [8]. In addition, PCV2 is capable of cross-species transmission and is associated with multiple diseases in cattle (including respiratory disease, a fatal hemorrhagic syndrome, and bovine neonatal pancytopenia). Globally, PCV2 infection has caused serious economic losses to the worldwide swine industry in the past fifty years.

Recently, a new species of the circovirus genus, PCV3, was detected in pigs with PDNS and PMWS in the USA [9], and subsequently in Poland, Germany, Brazil, and Italy [10–13]. In addition, this virus has become prevalent in many provinces and specific cities in China [14, 15]. Retrospective studies indicated that PCV3 infection could be traced back to 1996 [16]. These data suggest that PCV3 is an emerging and important pathogenic virus for pigs, with a worldwide distribution.

PCV3 has been implicated in a range of diseases. High positive rates (85.7%, 12/14) of PCV3 were reported in pigs suffering from reproductive failure [9, 17, 18]. Currently, most researchers recognize that PCV3 is classified into two genotypes, PCV3a and PCV3b [19–21]. Recently, Jiang et al. confirmed a PDNS-like clinical disease reproduced by PCV3 infection alone, and further research suggested that PCV3 is more pathogenic for piglets than PCV2 [22]. Surprisingly, recent works indicated that dogs could be infected by PCV3 [23]. The results indicated that PCV3 could transmit to non-porcine hosts, possibly through cross-species transmission routes. Consequently, we became interested in understanding the seroprevalence of PCV3 in cattle (*Bos taurus*), preferably using a molecular approach that facilitates any necessary genetic analyses.

## Results

### Screening for PCV3 prevalence in clinical samples

**Fig. 1:**
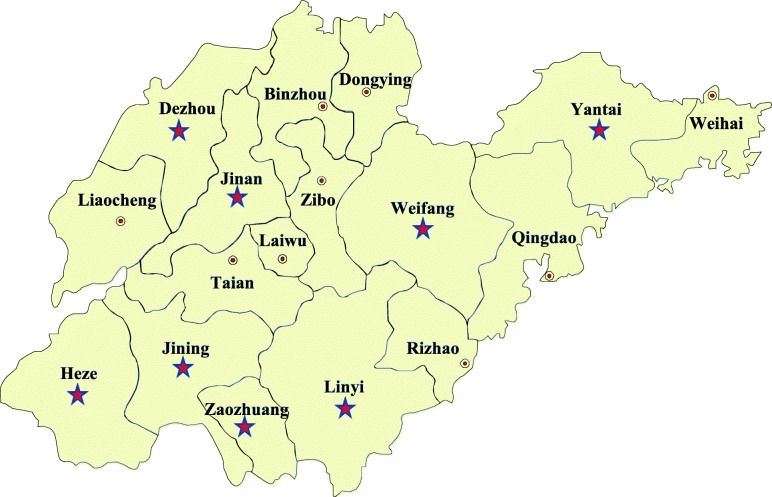
Geographical information for serum samples collected in Shandong Province. Red stars indicate the geographical location of the sample

In this study, among the 213 bovine serum samples, 74 were detected as positive for PCV3, representing a 34.7% positive detection rate. In addition, samples from11 of the 13 (84.6%) cattle farms in Shandong province were positive for PCV3. The rates were 34.6% (9/26) positivity in Jinan, 32.3% (11/34) positivity in Dezhou, 44.4% (12/27) positivity in Weifang, 30.0% (6/20) positivity in Linyi, 29.6% (8/27) positivity in Zaozhuang, 53.1% (17/32) positivity in Yantai, 27.3%(6/22) positivity in Heze, and 20.0% (5/25) positivity in Jining (Fig. 1).

### Multiple sequence alignment and analysis

Similar to the PCV2 genome, which has multiple sizes, the complete genome of PCV3 has been detected as two sizes: 1999 bp and 2000 bp [15, 19]. However, the four complete genomes (length = 2000 bp) sequenced in this study were similar to most previously isolated strains. In addition, 12 sequences of the capsid gene were determined. Multiple sequence alignment of these sequences showed 98.0–100% identity to the reference capsid gene sequence and 97.5–99.8% identity to the reference genome sequence. The alignment of the sequences among the PCV3 strains in this study showed that they shared 99.4 to 100% and 99.6 to 99.8% nucleotide similarity for the ORF2 gene and the complete genome, respectively.

**Fig. 2:**
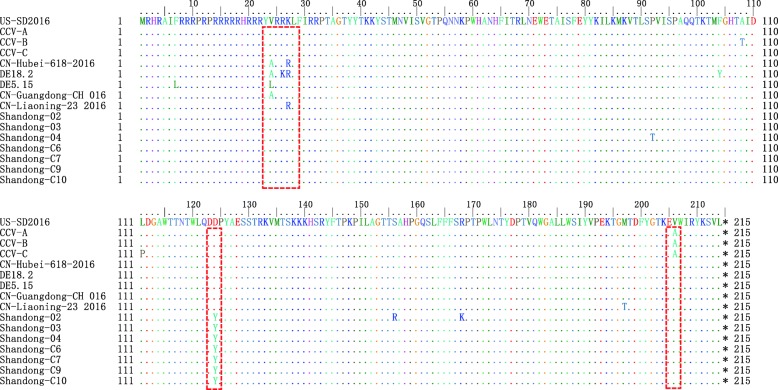
Alignment of amino acid sequences of capsid proteins representing different isolates of PCV3

Similar to previous reports, we detected six variant sequences of amino acids 24 to 27 of the Cap (VRRK, VRRR, ARRK, ARRR, ARKR, and LRRK); the vast majority of PCV3 strains are VRRK, with only the DE18.2 strains having ARKR and the DE5.15 strains having LRRK. Interestingly, a mutation was detected for the virus detected from cattle, compared with that isolated from pigs or dogs. Specifically, the inferred amino acid at position 124 (aspartic acid, D124) of the capsid protein detected in swine was mutated to tyrosine (Y124) in the virus detected in cattle (Fig. 2).

### Phylogenetic analysis of PCV3

**Fig. 3:**
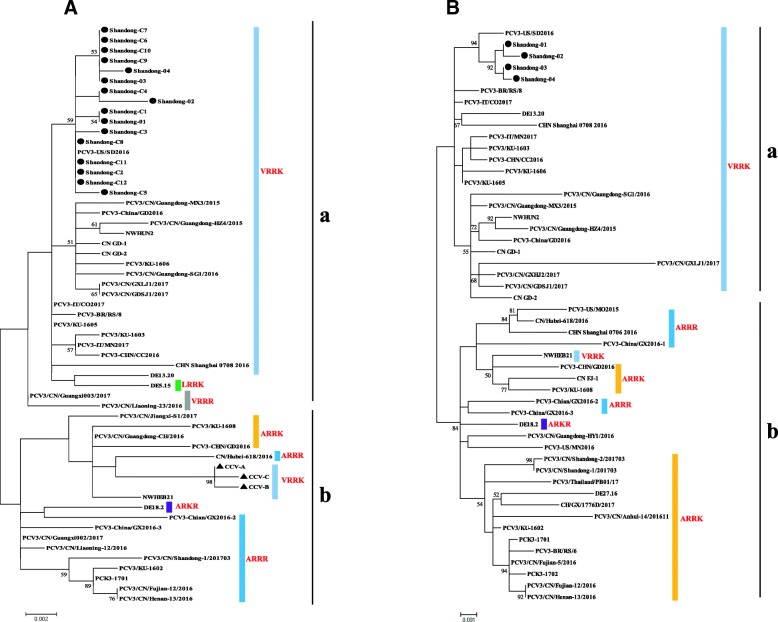
Phylogenetic analyses of capsid gene sequences **(a)** and complete genome sequences **(b)** from PCV3. The Maximum Likelihood (ML) trees were built using 1000 bootstraps replicates. Black circles indicate the strains detected in this study and black triangles indicate strains isolated from dogs. Others represent strains isolated from pigs

Based on their phylogenetic divergence, all the PCV3 strains can be classified into two different genotypes (PCV3a and PCV3b), and the strains in the present study were clustered in a branch representing PCV3a. Specifically, strains Shandong-01, 02, 03, and 04 had a close relationship to strain US/SD-2016, rather than strains detected in other parts of China (Fig. 3). The results revealed that PCV3a was prevalent in cattle in Shandong.

## Discussion

All types of farm animals (e.g., goats, cattle, camels, and chickens) and farm worker can be infected by circoviruses [2, 24, 25]. PCV2 is an important member of this family and is one of the major agents causing PCVAD, resulting in serious economic and production losses in the porcine industry [3–8]. PCV2 is capable of cross-species transmission to non-porcine hosts (for example, cattle, goats, rodents, and humans). PCV3 was first reported in 2016 and was detected in pigs with unexplained cardiac and multi-organ inflammation in the USA [9]. Recent research has confirmed that PCV3 is associated with multiple clinical diseases in infected pigs. Retrospective studies also showed that PCV3 strains collected between 1996 and 2017 had a high degree of genetic stability [16]. Surprisingly, recent works indicated that dogs could be infected by PCV3 [23], which suggested that the virus might also be propagated in hosts other than its natural host (pigs). These observations prompted us to speculate that similar to PCV2, PCV3 could be transmitted to cattle. In this study, the molecular epidemiological investigation results confirmed that PCV3 was prevalent in cattle in Shandong province, China.

PCV3 had been reported to be prevalent in at least seven countries, including America, Poland, German, and Italy [10, 12]. Especially, the prevalence of this virus was extensively reported in China, including many provinces and specific cities. Recent epidemiological surveillance data showed that a rate of PCV3 infection between 19.1 and 39.4% in different provinces (or cities) of China [14–16, 23]. In the present study, limited numbers of samples for detection may have resulted in the high prevalence in Yantai (53.1%) and low prevalence in Jinning (20%). Overall, the positive rates of PCV3 were 34.7% (74/213) in cattle in Shandong province, China.

Currently, there is a debate concerning the classification of PCV3. Overall, most research has divided PCV3 into two genotypes [14, 16, 18], from which, Li et al. used maximum likelihood (ML), maximum clade credibility (MCC), and neighbor joining (NJ) methods to reconstruct the phylogenies of PCV3 complete coding sequences, which were stably divided into two clades [20, 21]. In the present study, we used the methods detailed by Li et al., which also divided PCV3 into two different genotypes, PCV3a and PCV3b. At the same time, by combining genetic evolution analysis with amino acid sequence analysis, it was found that the 24th to the 27th amino acid sequence VRRR and most of VRRK were found mainly in PCV3a, and the amino acid sequences ARRK, ARKR, and ARRR are found mainly in PCV3b (Fig. 3). However, we encountered the same problem that adding more reference sequences for the ORF2 gene led to non-identical phylogenies. Therefore, we suggest using the whole genome sequences for PCV3 genotyping.

In circoviruses, Cap is not only the sole structural protein, but also contains immunologically important epitopes associated with virus neutralization. Therefore, it has been the main target for vaccine and diagnostic test development [4]. Multiple commercial vaccines against PCV2 have been introduced worldwide, which have been considered as a success story in veterinary vaccinology. However, the Cap of PCV3 and PCV2 share only approximately 30% amino acid identity [9]. Thus, cross protection seems unlikely. In previous reports, the amino acid at position 206 of the Cap mutated from lysine (V) to alanine (A) in dogs [23]. A similar mutation has been reported previously. Tyrosine at position 124 (Y124) of the Cap was only found in the bovine-origin PCV3 strains, whereas D124 was strictly conserved among all reference strains derived from dogs and pigs, based on all entries in the GenBank database. However, the limited reference sequence information in public databases makes it difficult to determine whether residue Y124 is truly a genetic marker to differentiate bovine-origin PCV3 from pig or dog-origin PCV3; thus, further research is required.

## Conclusion

This study reports is the first detection of PCV3 in cattle. Our results and those of previous reports indicate the possible transmission of PCV3 to non-porcine hosts, which might involve cross-species transmission. Notably, a comparison of the inferred amino acid sequences revealed a mutation from D124 to Y124 in the Cap. Further research is needed to determine the prevalence and pathogenesis of this virus in cattle, which would be useful to determine the possible origin and transmission route.

## Methods

### Sample information

From 2015 to 2017, serum samples (*n* = 213) of cows without clinical symptoms were collected from 13 cattle farms in Jinan, Yantai, Weifang, Linyi, Zaozhuang, Jining, Heze, and Dezhou in Shandong Province, China (Fig. 1). This study received animal ethics approval (No. Xidakezi2000138) from Guangxi University (see *Ethics approval and consent to participate*).

### DNA isolation and polymerase chain reaction (PCR)

DNA was extracted from these serum samples using a TIANamp Virus DNA Kit (TIANGEN, Beijing, China) according to the manufacturer’s instructions. Two primer pairs were designed based on sequences of the Chongqing-148/2016 strain (Accession no. KY075991.1) (Table 1). The PCR reaction mixture contained 1.5 μL of extracted DNA, 1 μL of primer pairs (10 μM), 12.5 μL PCR Master Mix (TIANGEN), and 10 μL of RNase-free water. The PCR amplification conditions were as follows: predenaturation at 94 °C for 5 min; followed by 35 cycles of denaturation at 94 °C for 30 s, annealing at 65 °C for 30 s and extension at 72 °C for 1 min; and then a final extension at 72 °C for 5 min. The PCR products were separated using a 1% agarose gel for purification, and then inserted into the pMD19-T vector after gel extraction. The recombined vectors were amplified in *Escherichia coli* DH5α and extracted for sequencing.

**Table 1:** List of primer sequences used in this study

Primer name	Sequence	Primer positions (nt)	Product size	Function
Cap-F	5’-TTACTTAGAGAACGGACTTGTAACG-3’	1339–1363	627	A
Cap-R	5’-AAATGAGACACAGAGCTATATTCAG-3’	1987–1965		A
PCV3-1F	5’-CAGCAGCTCGGATTCTGACGGAGAC-3’	560–584	1067	B
PCV3-1R	5’-TCCAAGACGACCCTTATGCGGAAAG-3’	1602–1626		B
PCV3-2F	5’-ACGGACTTGTAACGAATCCAAACTT-3’	1350–1374	1321	B
PCV3-2R	5’-CCCGCCCATAGCGTATAAATACTGC-3’	646–670		B

A: For the detection of PCV3 and sequencing full-length ORF2 gene sequence

B: For sequencing complete genome sequence

### Multiple sequence alignment and phylogenetic analysis

The ORF2 gene and the genome sequences of PCV3 obtained in this study have been deposited in GenBank under the accession numbers MH107148–MH107159 and MH107161–MH107165, respectively. Reference genome sequence for PCV3 was obtained from NCBI (Table 2). Multiple sequence alignments were carried out using ClustalW within the Megalign program (DNAStar software) and the phylogenetic relationships were determined using the maximum likelihood method and visualized with the MEGA software (version 7). Support for the phylogenetic relationships was determined by bootstrapping 1000 replicates. In the present study, the method described by Li et al. was used to divide clades of PCV3 [19–21].

**Table 2:** Summary of reference sequenced used in this study

Strain name	Accesion numbei	Country	Host	Complete genome or capsid gene
CCV-A	KY363870.1	China	canine	Capsid
CCV-B	KY363871.1	China	canine	Capsid
CCV-B	KY363872.1	China	canine	Capsid
PCV3/CN/Guangdong-CH/2016	MF589112.1	China	Pig	Capsid
PCV3/CN/Guangxi002/2017	MF374971.1	China	Pig	Capsid
PCV3/CN/Guangxi003/2017	MF374972.1	China	Pig	Capsid
PCV3/CN/Jiangxi-S1/2017	MF589133.1	China	Pig	Capsid
PCV3/CN/Liaoning-12/2016	KY354047.1	China	Pig	Capsid
PCV3/CN/Liaoning-23/2016	KY354055.1	China	Pig	Capsid
CH/GX/1776D/2017	MG550107.1	China	Pig	Complete
CHN_Shanghai_0706_2016	KY865242.1	China	Pig	Complete
CHN_Shanghai_0708_2016	KY865243.1	China	Pig	Complete
CN/Hubei-618/2016	KY354039.1	China	Pig	Complete
CN_FJ-1	KY753912.1	China	Pig	Complete
CN GD-1	KY753911.1	China	Pig	Complete
CN_GD-2	KY753913.1	China	Pig	Complete
NWHEB21	MG564174.1	China	Pig	Complete
NWHEB2	MG564175.1	China	Pig	Complete
PCV3/CN/Anhui-14/201611	MF084994.1	China	Pig	Complete
PCV3/CN/Fujian-12/2016	KY075987.1	China	Pig	Complete
PCV3/CN/Fujian-5/2016	KY075986.1	China	Pig	Complete
PCV3/CN/GDSJ1/2017	MF405271.1	China	Pig	Complete
PCV3/CN/Henan-13/2016	KY075988.1	China	Pig	Complete
PCV3/CN/Guangdong-HY1/2016	MF589102.1	China	Pig	Complete
PCV3/CN/Guangdong-HZ4/2015	MF589103.1	China	Pig	Complete
PCV3/CN/Guangdong-MX3/2015	MF589104.1	China	Pig	Complete
PCV3/CN/Guangdong-SG1/2016	MF589105.1	China	Pig	Complete
PCV3/CN/GXHJ2/2017	MF405277.1	China	Pig	Complete
PCV3/CN/GXLJ1/2017	MF405276.1	China	Pig	Complete
PCV3/CN/Shandong-1/201703	KY778776.1	China	Pig	Complete
PCV3/CN/Shandong-2/201703	KY778777.1	China	Pig	Complete
PCV3-Chian/GX2016–2	MF155642.1	China	Pig	Complete
PCV3-China/GD2016	KY418606.2	China	Pig	Complete
PCV3-China/GX2016–1	MF155641.1	China	Pig	Complete
PCV3-China/GX2016–3	MF155643.1	China	Pig	Complete
PCV3-CHN/CC2016	KY421348.1	China	Pig	Complete
PCV3-CHN/GD2016	KY421347.1	China	Pig	Complete
PCV3/Thailand/PB01/17	MG310152.1	Thailand	Pig	Complete
PCV3-IT/CO2017	MF162298.1	Italy	Pig	Complete
PCV3-IT/MN2017	MF162299.1	Italy	Pig	Complete
PCV3-US/MN2016	KX898030.1	USA	Pig	Complete
PCV3-US/MO2015	KX778720.1	USA	Pig	Complete
PCV3-US/SD2016	KX966193.1	USA	Pig	Complete
DE5.15	MG014378.1	Germany	Pig	Capsid
DE13.20	MG014365.1	Germany	Pig	Complete
DE18.2	MG014365.1	Germany	Pig	Complete
DE27.16	MG014370.1	Germany	Pig	Complete
PCK3–1701	MF611876.1	South Korea	Pig	Complete
PCK3–1702	MF611877.1	South Korea	Pig	Complete
PCV3/KU-1602	KY996338.1	South Korea	Pig	Complete
PCV3/KU-1603	KY996339.1	South Korea	Pig	Complete
PCV3/KU-1605	KY996341.1	South Korea	Pig	Complete
PCV3/KU-1606	KY996342.1	South Korea	Pig	Complete
PCV3/KU-1608	KY996344.1	South Korea	Pig	Complete
PCV3-BR/RS/6	MF079253.1	Brazil	Pig	Complete
PCV3-BR/RS/8	MF079254.1	Brazil	Pig	Complete

## Abbreviations

A: Alanine
Cap: Capsid protein
D: Aspartic acid
K: Lysine
L: Leucine
MCC: Maximum clade credibility
ML: Maximum likelihood
NJ: Neighbor joining
ORF: Open-reading frame
PCV: Porcine circovirus
PCVAD: PCV-associated disease
PCVD: Porcine circovirus disease
PDNS: Porcine dermatitis and nephropathy syndrome
PMWS: Post-weaning multi-systemic wasting syndrome
R: arginine
Rep: Replication-associated protein
V: Lysine
Y: Tyrosine
